# Assessment of Lateral Abdominal Muscle Activation Asymmetry via M-mode Ultrasonography

**DOI:** 10.7759/cureus.57563

**Published:** 2024-04-03

**Authors:** Maciej Biały, Wojciech Szewczyk, Patryk Kłaptocz, Rafał Gnat

**Affiliations:** 1 Physiotherapy, Institute of Physiotherapy and Health Sciences, The Jerzy Kukuczka Academy of Physical Education, Katowice, POL; 2 Physiotherapy, Functional Diagnostics Laboratory, Sport-Klinika, Scanmed Sport, Żory, POL; 3 Physiotherapy, Shoulder and Knee Clinic, Katowice, POL; 4 Radiology, Helimed Diagnostic Imaging, Poland, POL

**Keywords:** postural balance, m-mode ultrasonography, tissue deformation index, asymmetry, lateral abdominal muscle

## Abstract

Introduction: This study aimed to evaluate the asymmetry in the lateral abdominal muscles (LAMs) expressed as tissue deformation index asymmetry (aTDI) with the use of M-mode ultrasonography. The muscles of interest were the transversus abdominis, internal oblique, and external oblique.

Methods: This is a cohort of 126 healthy subjects who participated in the study. Measurements were taken by two raters, blinded to the aim of the study. M-mode ultrasounds with a measurement frequency of 5 MHz were utilized to record the postural response of LAMs to external perturbation in the form of rapid arm abduction with load, and individual aTDI values for each muscle were calculated.

Results: The aTDI values from deep to superficial LAMs were 78.28% for transversus abdominis, 55.68% for internal oblique, and 44.80% for external oblique. Only the aTDI for transversus abdominis results differed significantly from those of the other LAMs (P<0.05).

Conclusion: LAM asymmetry values exhibit the following gradient: transversus abdominis >internal oblique >external oblique. Specifically, only transversus abdominis demonstrates noteworthy asymmetry in postural activity. This observation contributes to the literature by indicating that transversus abdominis asymmetry may serve as a marker for assessing the variability in motor control of the deep abdominal musculature. The dominance of transversus abdominis (TrA) asymmetric activity underlines the importance of personalized approaches for patients with lumbopelvic disorders or for athletes seeking to enhance performance.

## Introduction

Magnetic resonance imaging (MRI) is considered a gold standard for non-invasive assessment of muscle thickness and has a high correlation with cadaver muscle measurement [1]; however, there are some limitations to this technique, e.g., financial costs, accessibility, and a restricted measurement position [2]. Alternatively, low-cost real-time musculoskeletal ultrasonography (US) has made significant improvements in the scientific field in recent decades. It has been proven that changes in US muscle cross-sectional area correspond with changes in MRI muscle thickness [3] and electromyographic muscle activity [4], which makes this technique a valuable tool for the diagnosis and treatment of musculoskeletal disorders (e.g., as a biofeedback device in physiotherapeutic exercises) [5].

One of the muscle groups most explored in vivo with the use of US is the lateral abdominal muscle (LAM) group [6]. Postural co-activation of the LAMs as a response to internal and external forces is essential for lumbar spine and trunk stabilization [7-10].­ This muscular unit is functionally divided into the following two layers: deep layer, consisting of transversus abdominis (TrA), and superficial layer, consisting of external oblique (EO) with internal oblique (IO). LAMs are commonly involved in breathing [11], postural control [7], and multidirectional movements during various activities [12]. Asymmetry in LAM thickness between the left and right sides of the body has been reported in healthy subjects [13], low back pain patients [14], and athletes [15]. A theory that explains the potential negative influence of LAM asymmetry indicates that repetitive asymmetrical torque can cause inflammation of the intervertebral disc and facet joints [16]. Nevertheless, as mentioned earlier [7-16], the asymmetrical action of LAMs can be observed in different populations without signs of any visible pathology, and the level of activation among each LAM may also differ according to the intensity and type of activity performed [17].

Recently, a reliable measurement (intra-class correlation coefficient {ICC} >0.8) of the reflex response of LAMs to postural disturbance involving M-mode real-time US imaging was introduced and verified in a healthy population [18,19], as well as in subjects with experimentally induced low back pain [20]. The method is based on the calculation of the so-called tissue deformation index (TDI) and illustrates the percentage change in the given LAM thickness over time. It has been found that the TDI values of LAMs are characterized by a specific gradient of muscle deformation: TrA<IO<EO, which can be observed regardless of body side [18].

This study aimed to evaluate the asymmetry in TDI (aTDI) of the individual LAMs (right versus left body side) and compare the differences in aTDI between TrA, IO, and EO. Also, this study aimed to provide the normative data on aTDI for further comparison with the clinical population, only healthy subjects were engaged in the study.

## Materials and methods

This was a prospective study involving 126 (59 females) subjects (mean age: 22.87±2.61 years, body mass index: 22.90±2.46 kg/m^2^). All participants were checked against the following inclusion criteria: no history of pain requiring medical intervention, injury, or surgery within the lumbopelvic area; no functional defects of the upper extremities; and well-being on the measurement day. Additionally, subjects who participated in regular exercise training engaging the lumbar and abdominal musculature were excluded. All subjects signed a written informed consent. This study was conducted following the principles of the Declaration of Helsinki, and approved by the Ethical Committee of the Jerzy Kukuczka Academy of Physical Education, Katowice, Poland (#18/2007). All measurements were performed by two raters blinded to the objective of the study. In order to supervise the measurement procedure, a third independent rater was also involved.

**Figure 1:**
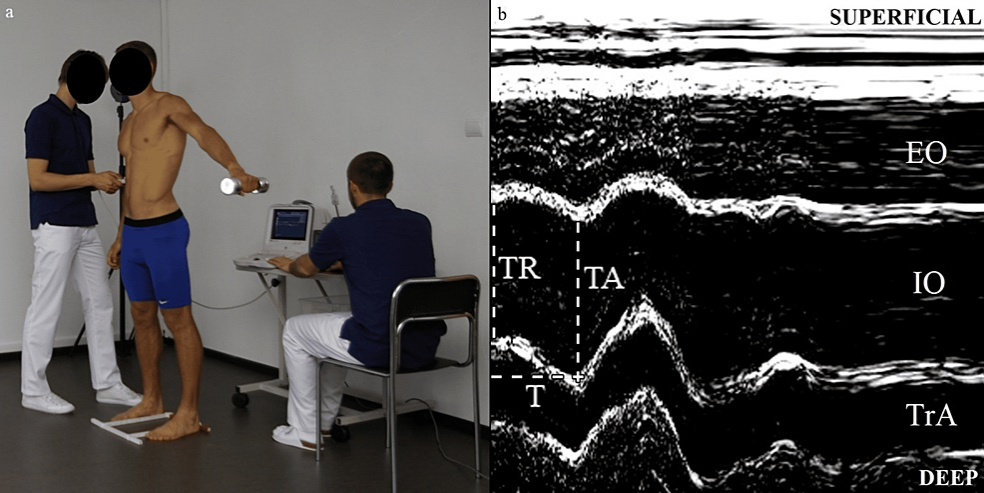
M-mode ultrasonographic measurements capturing lateral abdominal muscle activation during postural perturbation induced by rapid contralateral arm abduction. Rapid contralateral arm abduction with a weight of 3 kg (a). An example of measurements performed on the oblique internal muscle are presented (b). TR: muscle thickness at rest; TA: the thickness at maximal activation; T: time to maximal activation; EO: external oblique muscle; IO: internal oblique muscle; TrA: transversus abdominis muscle

Ultrasound evaluation was performed using the Mindray DP 6600 US unit (Shenzhen, China: Mindray) with the linear array. With the array placed horizontally, Rater 1 randomly selected one side of the body to determine the ideal position for imaging the LAMs. Starting from the navel, the array was gradually shifted laterally until a clear image of the three layers of the LAMs appeared on the screen. The precise array location was marked on the skin using elastic Kinesio tape with an opening that matched the array shape, allowing for easy reproducibility of the array's position if needed. Subsequently, US device was switched to M-mode, and LAM deformation in response to postural perturbation provoked by rapid arm abduction with a load of 3 kg was recorded. Each movement was triggered by an auditory stimulus synchronized with the start of M-mode image registration, due to measurement character and possibility of visual disturbances during assessment, a frequency of 5 MHz was set (Figure 1, panel a) [18]. Six repetitions of arm abduction (up to 90°) were performed, and six M-mode US images were gathered for each body side. In total, 12 US images of the LAMs were gathered from each subject. After image quality verification, the four poorest images were excluded. For data extraction, Photoshop 8.0 software (San Jose, CA: Adobe) was used, and for each LAM, the following measurements were taken: muscle thickness at rest (TR [mm]), muscle thickness at the point of maximal activation (TA[mm]) and time to achieve maximal activation (T[ms]) (Figure 1, panel b).

For each individual LAM, the TDI (%/ms) was calculated with the use of the following formula: [(TA/TR × 100%) - 100%] × T-1. To calculate the percentage asymmetry in LAMs between body sides, the following modified formula proposed by Kim et al. was implemented: aTDI = [(TDIr - TDIl)/(1/2) (TDIr + TDIl)] × 100%, where aTDI is tissue deformation index asymmetry (%), TDIr is tissue deformation index for the right body side (%/ms), and TDIl is tissue deformation index for the left body side (%/ms) [21]. The aTDI is the absolute difference in individual LAMs TDI between the left and right sides normalized to the sum of TDIs on both sides. The lower the aTDI values, the lower the asymmetry observed between body sides. The presented procedure for LAM measurement has been tested in a pilot study and its reliability has been reported previously with ICC values for TrA of 0.81; IO of 0.88 and EO of 0.87 [18,19].

Statistical analysis was performed in Statistica 10 software (Tulsa, OK: StatSoft Inc.). To verify the distributions, normality analysis was carried out using the Shapiro-Wilk test. Kruskal-Wallis ANOVA was implemented to test for differences in aTDI between the TrA, IO, and EO, together with its own post hoc test for multiple comparisons. The alpha level of 0.05 was considered significant.

## Results

**Figure 2:**
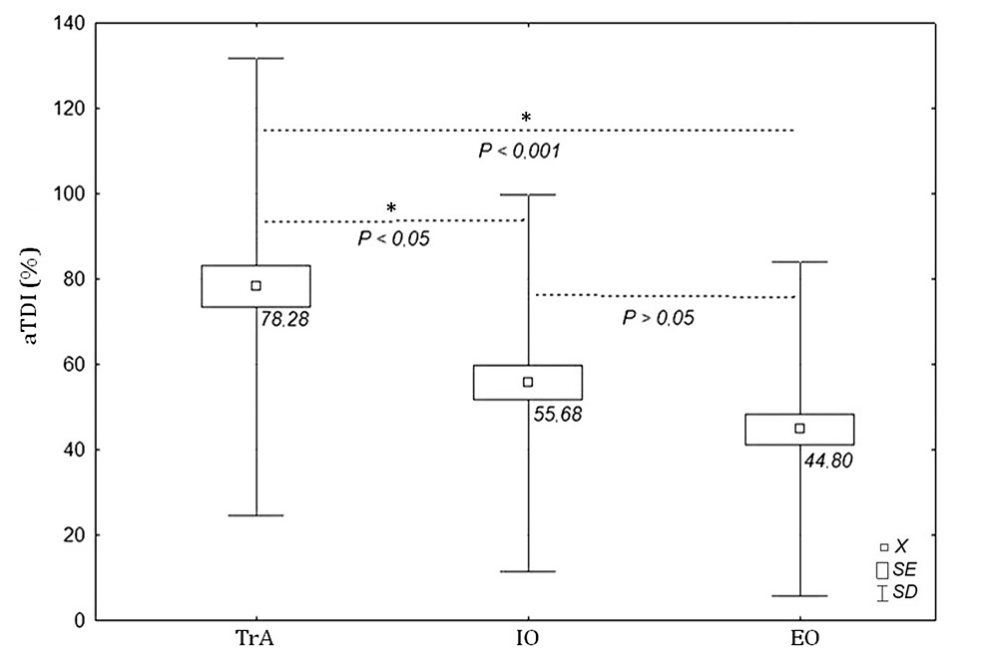
Values of tissue deformation index asymmetry (aTDI {%}) for lateral abdominal muscles (TrA, IO, EO) regardless of body side. *P<0.05 and <0.001 were considered statistically significant. Squares denote mean value (X), frames denote the standard error (SE), and whiskers denote the standard deviation (SD). TrA: transversus abdominis; IO: internal oblique; EO: external oblique

We observed statistically significant intra-muscle differences (TrA vs IO vs EO) in mean aTDI values (ANOVA: H=26.80, P<0.001). In post hoc analysis, the highest average aTDI values of 78.28±53.57%, 95% CI [68.56 ÷ 88.01] were registered for TrA, 55.68±44.13%, 95% CI [47.80 ÷ 63.56] for IO, and 44.80±39.19%, 95% CI [37.83 ÷ 51.77] for EO. Detailed aTDI results for individual LAMs are presented in Table 1. For TrA muscle, aTDI values were significantly higher compared with those for IO (P<0.05) and EO (P<0.001). No significant differences between IO and EO were recorded (P>0.05) (Figure 2).

**Table 1:** Tissue deformation index asymmetry for individual lateral abdominal muscles. aTDI: tissue deformation index asymmetry; TrA: transversus abdominis; IO: internal oblique; EO: external oblique; Min.: minimal value; Max.: maximal value

Parameter (n=126)	Mean±SD	-95% CI	+95% CI	Min.	Max.
aTDI (%)					
TrA	78.28±53.57	68.56	88.01	1.54	193.72
IO	55.68±44.13	47.80	63.56	0.45	232.83
EO	44.80±39.19	37.83	51.77	0.01	185.01

## Discussion

The differences in the aTDI values of LAMs demonstrate a characteristic pattern in which the aTDI is highest for the deep muscles and gradually decreases towards the superficial layers. The highest mean aTDI value of 78.28±53.57% was that of the TrA muscle and was significantly different from that of the IO (55.68±44.13%) and EO (44.8±39.19%).

These results are in line with our previous findings on the TDI gradient of LAMs (TrA<IO<EO), in which the deepest TrA yielded the lowest value (0.06%/ms), IO (0.11%/ms) and EO (0.16%/ms) and can be explained by the following LAM morphology: deep muscles consists mainly of slow-twitch fibres (type I) in contrast to more superficial units [18], which are mainly built from fast-twitch fibers (type II) [22]. It is noticeable that individual LAM asymmetry also manifests itself as a gradient having the greatest average value for TrA and gradually decreasing towards EO (aTDI: TrA {78.28%}>IO {55.68%}>EO {44.80%}).

It should be emphasized that we measured a healthy, homogenic group, and every subject passed the inclusion criteria. Considering previously reported normative data for LAMs thickness, the symmetrical pattern was to be expected [23]. However, TrA activation was characterized by a significant asymmetry of 78.28%. These phenomena were not observed so clearly for OI and OE. Deep abdominal muscles are considered to be critical for lumbar spine segmental stability and preventing overloading and injury of passive structures [24]. This TrA protection mechanism relies on increasing intra-abdominal pressure and mainly, through its connection with the lumbar fascia, increases the stiffness of the lumbar spine [25]. Thus, symmetry in activity should provide "symmetrical protection" for the lumbar spine. However, it seems that TrA on each side of the body acts independently, in a more "sophisticated" manner, and rather depends on the movement task and/or external environment (e.g., the subject’s body position (sitting vs standing) during the measurement procedure), which stands in contrast to features of the more superficial motor units [26]. Perhaps random and asymmetrical TrA activity should be considered as a physiological attribute of this muscle, and the presented results recorded with US are a manifestation of its unique motor control pattern. This hypothesis might be explained by a study presented by Gnat et al., who analyzed brain activity during deep and superficial LAM contraction and found that TrA activity is more difficult to coordinate and engages the brain in a more complex way than activation of IO and EO [27]. TrA asymmetry may be an expression of different TrA motor control and random activity compared to that of other more superficial LAMs. This statement coincides with the results presented by other authors. A greater variability in the deep LAM response to postural perturbations was found among healthy subjects in comparison to patients with low back pain [27]. This phenomenon was also observed in 20% of young males [28]. Allison et al. arrived at the conclusion that anticipatory TrA activation is characteristic exclusively of the muscles located contralaterally to the upper extremity that produce postural perturbation [29]. These results directly correspond with our measurement technique, in which we evaluated TDI asymmetry of LAMs as a postural response to the movement of contralateral arm abduction. During arm flexion or extension, there is a possibility that rotation torque will influence TrA activation because of forces parallel to the muscle fibers. Moreover, we found that images recorded during arm abduction characterized sufficient graphical quality. The presented study has several limitations. Firstly, our results are confined to a healthy and predominantly young cohort; however, they hold clinical utility as they establish a valuable reference for symptomatic populations. Additionally, subjective and arbitrary image contrast adjustments during image analysis may introduce errors, as inappropriate adjustments could distort perceived muscle boundaries despite being tested in previous studies [18]. Moreover, standardized control of the ultrasound array position and pressure was not provided, which was particularly problematic during rapid arm abduction; nevertheless, we believe this limitation can be mitigated through adequate training of the raters.

## Conclusions

This study provides novel insights into LAMs activity asymmetry, revealing a TrA>IO>EO gradient. Notably, the TrA muscle exhibits significantly higher asymmetry in postural activity, indicative of deep musculature motor control complexity. The utilization of M-mode ultrasound for LAMs asymmetry evaluation offers valuable clinical reference data, particularly for populations such as those with low back pain. Unlike previous research focusing on voluntary muscle activation, this study comprehensively compares key abdominal muscles' postural activity within the same cohort, combining precision and reliability with the simplicity of the measurement technique. These findings advance the understanding of abdominal muscle function and provide a basis for tailored therapeutic interventions to optimize trunk motor control and performance.
